# Quality assessment of the MRI-radiomics studies for MGMT promoter methylation prediction in glioma: a systematic review and meta-analysis

**DOI:** 10.1007/s00330-024-10594-x

**Published:** 2024-02-03

**Authors:** Fabio M. Doniselli, Riccardo Pascuzzo, Federica Mazzi, Francesco Padelli, Marco Moscatelli, Tugba Akinci D’Antonoli, Renato Cuocolo, Domenico Aquino, Valeria Cuccarini, Luca Maria Sconfienza

**Affiliations:** 1 Neuroradiology Unit, Fondazione IRCCS Istituto Neurologico Carlo Besta, Via Giovanni Celoria 11, 20133 Milan, Italy; 2 Department of Biomedical Sciences for Health, Università degli Studi di Milano, Via Luigi Mangiagalli 31, 20133 Milan, Italy; 3 Institute of Radiology and Nuclear Medicine, Cantonal Hospital Baselland, Rheinstrasse 26, 4410 Liestal, Switzerland; 4 Department of Medicine, Surgery, and Dentistry, University of Salerno, Via Salvador Allende 43, Baronissi, 84081 Salerno, Italy; 5 IRCCS Ospedale Galeazzi-Sant’Ambrogio, Via Cristina Belgioioso 173, 20157 Milan, Italy

**Keywords:** Glioma, O(6)-Methylguanine-DNA methyltransferase, Magnetic resonance imaging, Systematic review, Meta-analysis

## Abstract

**Objectives:**

To evaluate the methodological quality and diagnostic accuracy of MRI-based radiomic studies predicting O6-methylguanine-DNA methyltransferase (MGMT) promoter methylation status in gliomas.

**Methods:**

PubMed Medline, EMBASE, and Web of Science were searched to identify MRI-based radiomic studies on MGMT methylation in gliomas published until December 31, 2022. Three raters evaluated the study methodological quality with Radiomics Quality Score (RQS, 16 components) and Transparent Reporting of a Multivariable Prediction Model for Individual Prognosis Or Diagnosis (TRIPOD, 22 items) scales. Risk of bias and applicability concerns were assessed with QUADAS-2 tool. A meta-analysis was performed to estimate the pooled area under the curve (AUC) and to assess inter-study heterogeneity.

**Results:**

We included 26 studies, published from 2016. The median RQS total score was 8 out of 36 (22%, range 8–44%). Thirteen studies performed external validation. All studies reported AUC or accuracy, but only 4 (15%) performed calibration and decision curve analysis. No studies performed phantom analysis, cost-effectiveness analysis, and prospective validation. The overall TRIPOD adherence score was between 50% and 70% in 16 studies and below 50% in 10 studies. The pooled AUC was 0.78 (95% CI, 0.73–0.83, *I*^2^ = 94.1%) with a high inter-study heterogeneity. Studies with external validation and including only WHO-grade IV gliomas had significantly lower AUC values (0.65; 95% CI, 0.57–0.73, *p* < 0.01).

**Conclusions:**

Study RQS and adherence to TRIPOD guidelines was generally low. Radiomic prediction of MGMT methylation status showed great heterogeneity of results and lower performances in grade IV gliomas, which hinders its current implementation in clinical practice.

**Clinical relevance statement:**

MGMT promoter methylation status appears to be variably correlated with MRI radiomic features; radiomic models are not sufficiently robust to be integrated into clinical practice to accurately predict MGMT promoter methylation status in patients with glioma before surgery.

**Key Points:**

• *Adherence to the indications of TRIPOD guidelines was generally low, as was RQS total score.*

• *MGMT promoter methylation status prediction with MRI radiomic features provided heterogeneous diagnostic accuracy results across studies.*

• *Studies that included grade IV glioma only and performed external validation had significantly lower diagnostic accuracy than others.*

**Supplementary Information:**

The online version contains supplementary material available at 10.1007/s00330-024-10594-x.

## Introduction

Gliomas are the most common primary malignant brain tumors in adults, with a median age of onset of approximately 55 to 60 years [1]. In high-grade gliomas, the use of temozolomide after gross total resection represents first-line medical therapy associated to radiation therapy [2, 3]. Among all identified glioma genetic alterations, O6-methylguanine-DNA methyltransferase (MGMT) promoter methylation is an important prognostic molecular marker in clinical settings[4].

The MGMT normally protects cells against the damage of alkylating agents [5] such as temozolomide, and therefore its inhibited expression through methylation is related to a better prognosis during standard-of-care chemotherapy for glioblastoma [2, 6, 7]. Indeed, MGMT methylation is associated with better survival, especially for patients with higher extent of MGMT methylation [8].

In a minority of patients, surgical resection is not possible (due to concomitant pathologies or old age) or tests for MGMT methylation are unsuccessful due to tissue insufficiency, especially for those undergoing stereotaxic biopsy. Therefore, the opportunity to determine MGMT methylation status through imaging without surgical intervention would be of great utility for patient management.

Several prior studies tried to correlate MRI data with MGMT methylation status, based on visual assessment of experienced radiologists. Results of these works were not always consistent, with alternate conclusions [9, 10]. The introduction of machine-learning methods based on the extraction of radiomic features has revitalized the debate [11, 12].

Radiomics is a research field that exploits the increased computing capabilities that have become available over the last few decades to extract and analyze thousands of quantitative biomarkers from radiological images [13]. Many research groups have been investigating the correlation of radiomic features with MGMT methylation status. Their findings vary greatly ranging from very promising predictive values [14] to disappointing results [15].

Radiomic studies have great variability in their methodological pipelines, which can impact the reproducibility and generalizability of results. In this context, the Image Biomarkers Standardization Initiative (IBSI) represents an international effort to provide standardized procedures for image processing and radiomic features calculation [16]. One of the methodological studies aiming to provide specific recommendations for reporting radiomic models is the Radiomics Quality Score (RQS) [17]. This tool proposes a standardized evaluation of the performance, reproducibility, and clinical utility of radiomic studies by assessing compliance not only with feature extraction but also with model development and validation. The RQS has been already applied to evaluate the methodological quality of radiomic studies for several oncological diseases such as meningiomas, gliomas, metastases, and other neoplasms [18–21].

This review aimed to evaluate the quality of prior studies on predicting MGMT methylation status in gliomas based on MRI-radiomic features, using the RQS items. We also assessed the studies using the Transparent Reporting of a Multivariable Prediction Model for Individual Prognosis Or Diagnosis (TRIPOD) guideline [22], a commonly used standard for reporting studies that develop and/or validate prediction models. Furthermore, we conducted a meta-analysis to quantitatively investigate the association between study quality and diagnostic accuracy while accounting for the heterogeneity of the included studies.

## Materials and methods

This study presents a systematic review of the literature and a meta-analysis conducted in compliance with the Preferred Reporting Items for Systematic Reviews and Meta-analysis (PRISMA) statement. Ethical committee approval was waived due to the nature of the study.

### Eligibility criteria

Studies were included if the following criteria were met: (1) aimed to predict MGMT methylation status using any MRI sequence; (2) used radiomic features as input for classification; (3) included patients with glioma of any WHO grade (I to IV, as defined according to the 2016 edition of the WHO central nervous system tumor classification [23]). Studies were excluded if (1) they were case reports, correlation studies, commentaries, conference abstracts, editorials, letters, and review articles, or (2) radiomic features did not encompass texture features as defined according to IBSI guidelines.

### Search strategy and study selection

We performed a systematic search in PubMed Medline, EMBASE, and Web of Science databases to identify any published study until 31 December 2022, using the following query: (glioma OR glioblastoma) AND (MGMT OR ‘Methylguanine methyltransferase’) AND (radiomic OR radiogenomic OR texture).

First, duplicated studies were removed. Then, studies were independently screened by three authors (F.M.D. and M.M. with 7 years and R.P. with 8 years of experience in neuroimaging research), first reviewing the titles and abstracts to determine whether they met exclusion criteria and should be removed. Studies found to be not eligible after title and abstract screening were excluded. Next, a full text review of the identified studies was performed to determine the final list of included studies. Disagreements were resolved by consensus.

### Data extraction

In a spreadsheet, we collected data on the number of patients included, WHO grade of tumors, additional molecular markers explored, number of MRI scanners, MRI sequences, number and type of tumor compartments segmented and considered for feature extraction (e.g., only contrast-enhancing areas, necrotic areas, T2/FLAIR hyperintense areas), number of extracted features, and methods for feature extraction and classification, as well as classification performances (i.e., area under the curve [AUC], accuracy).

### Quality assessment

We evaluated the methodological quality of the included studies based on the RQS and TRIPOD guidelines. Our focus was only for the MGMT prediction task and not for other tasks potentially performed in the same study. In addition, risk of bias and applicability concerns were assessed using the Quality Assessment of Diagnostic Accuracy Studies 2 (QUADAS-2), one of the most recommended tools for evaluating such risks in systematic reviews on diagnostic accuracy studies [24]. Details about QUADAS-2 are illustrated in the Supplementary Methods.

#### RQS

Two reviewers (F.M.D. and R.P.) achieved consensus on the evaluation criteria of RQS by discussion and independently evaluated the included studies by scoring each RQS item. Disagreements on the scores assigned to the studies were resolved by discussion and consensus or else by a third reviewer (M.M.) to avoid a potentially low inter-rater reproducibility as highlighted by recent evidence [25]. Next, we classified the 16 components of RQS into six domains, in line with previous studies [18, 19, 26, 27], and calculated the cumulative score for each domain of the RQS and overall. Table 1 reports the RQS components grouped by domain, with range of attainable points.

**Table 1:** Description of RQS items divided per domain, with scoring ranges and criteria

Domain 1—Protocol quality and stability in image and segmentation (0–5 points)	
a)	Image protocol quality (item 1)
	+ 1 (if protocols are well-documented)
	+ 1 (if public protocol is used)
b)	Imaging at multiple time points (item 4)
c)	Phantom study on all scanners—detect inter-scanner differences and vendor-dependent features (item 3)
d)	Multiple segmentations (item 2)
	+ 1 for each 1b, 1c, and 1d
Domain 2—Feature selection and validation (−8 to 8 points)	
a)	Feature reduction or adjustment for multiple testing (item 5)
	− 3 (if neither measure is implemented) + 3 (if either measure is implemented)
b)	Validation (item 12)
	− 5 (if validation is missing)
	+ 2 (if validation is based on a dataset from the same institute)
	+ 3 (if validation is based on a dataset from another institute)
	+ 4 (if validation is based on two datasets from two distinct institutes)
	+ 4 (if the study validates a previously published signature)
	+ 5 (if validation is based on three or more datasets from distinct institutes)
Domain 3—Biologic/clinical validation and utility (0–6 points)	
a)	Multivariable analysis with non-radiomics features (item 6)
b)	Detect and discuss biological correlates (item 7)
	+ 1 for each 3a and 3b
c)	Comparison to “gold standard” (item 13)
d)	Potential clinical utility (item 14)
	+ 2 for each 3a and 3b
Domain 4—Model performance index (0–5 points)	
a)	Cut-off analyses (item 8)
	+ 1 if cut-off analyses are conducted
b)	Discrimination statistics (item 9)
	+ 1 (if a discrimination statistic and its statistical significance are reported)
	+ 1 (if a resampling method technique is also applied)
c)	Calibration statistics (item 10)
	+ 1 (if a calibration statistic and its statistical significance are reported)
	+ 1 (if a resampling method technique is also applied)
Domain 5—High level of evidence (0–8 points)	
a)	Prospective study registered in a trial database (item 11)
	+ 7 (for prospective validation of a radiomics signature in an appropriate trial)
b)	Cost-effectiveness analysis (item 15)
	+ 1 if cost-effectiveness analyses are conducted
Domain 6—Open science and data (0–4 points)	
a)	Open science and data (item 16)
	+ 1 (if scans are open source)
	+ 1 (if region of interest segmentations are open source)
	+ 1 (if code is open source)
	+ 1 (if radiomics features are calculated on a set of representative ROIs and the calculated features and representative ROIs are open source)

*RQS* Radiomics Quality Score

#### TRIPOD

Two reviewers (F.M. and F.P.) achieved consensus on the evaluation criteria of TRIPOD by discussion and independently evaluated the included studies by scoring each TRIPOD item. Disagreements were resolved by discussion and consensus or else by a third reviewer (M.M.). A total TRIPOD adherence score was calculated for each study by summing the adhered and applicable TRIPOD items. Overall adherence per TRIPOD item was calculated by dividing the number of studies that adhered to a specific TRIPOD item by the number of studies in which the specific TRIPOD item was applicable [22].

### Statistical analysis

The measured endpoint was the AUC of each study obtained from models based only on radiomics (excluding clinical data). Of note, only studies that reported a measure of uncertainty of their AUC (either standard deviations or 95% confidence intervals [CIs]) were included in the meta-analysis. Authors of studies that did not report such information in their publications were contacted via email to ask for these missing data. Pooled results with corresponding 95% CIs were derived using the random effects model based on restricted maximum likelihood estimator [28, 29]. The heterogeneity of individual studies was estimated with Cochran’s *Q* test and *I*^2^ value. Sources of heterogeneity among studies were identified by performing subgroup analyses and meta-regression, considering the combination of the following two factors: (1) external validation was performed; (2) patients with only WHO-grade IV were included. Publication bias was assessed by funnel plot and Egger’s test [30]. The statistical analyses were performed using R (version 4.2.1) and, in particular, the “metafor” R package [31].

## Results

**Fig. 1:**
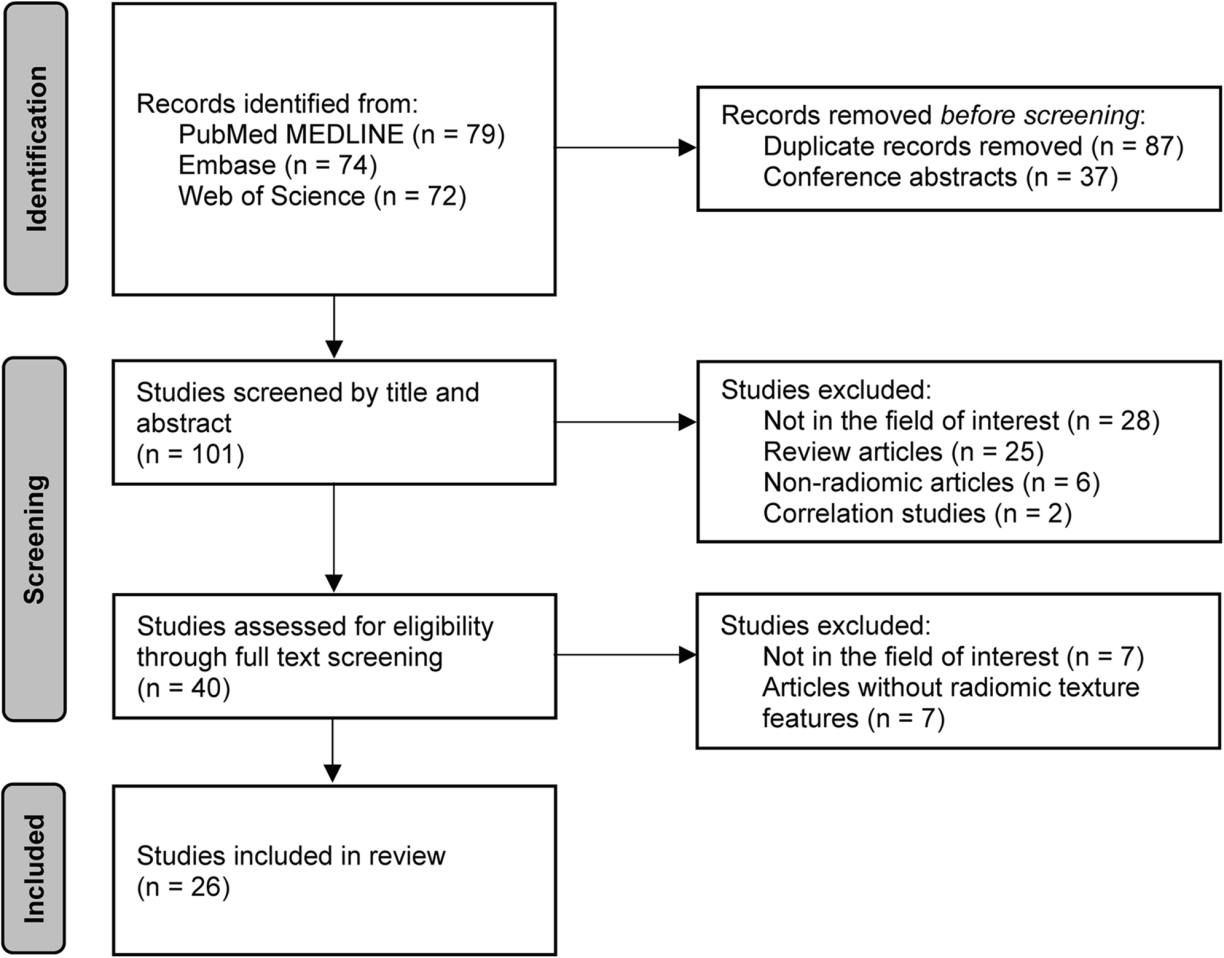
Study selection flowchart

We identified 101 studies, of which 26 met the inclusion criteria (Fig. 1, Table 2) [11, 12, 14, 15, 32–53]. These studies included a median of 116 patients (min 34; max 418). The majority (15/26, 58%) focused on patients with WHO grade IV gliomas, and the others included patients with heterogeneous WHO grades gliomas (from I to IV, or only II and III). Other information related to image and radiomic workflow is shown in Supplementary Table 1.

**Table 2:** List of the 26 studies included in this review. All studies had retrospective design

Ref	First author	Year	Journal	No. of patients	No. of patients in separate validation set	No. of scanner/magnetic fields	MRI sequences	WHO grade glioma
[32]	Calabrese	2022	Neurooncol Adv	381	–	1/3 T	3D T1, 3D T1-Gd, 3D FLAIR, 3D T2, SWI, ASL, DWI	IV
[33]	Chen	2022	J Clin Med	111	22	1/3 T	T1, T1-Gd, T2, FLAIR	II–IV
[34]	Crisi	2020	J Neuroimaging	59	–	1/3 T	DSC	IV
[35]	Do	2022	Sci Rep	53	–	TCIA	T1, T1-Gd, T2, FLAIR	IV
[36]	Hajianfar	2019	World Neurosurg	82	–	TCIA	T1-Gd, FLAIR	IV
[37]	Haubold	2021	Cancers (Basel)	164	33	7/1.5 T and 3 T	T1, T1-Gd, FLAIR	II–IV
[38]	Haubold	2020	Eur J Nucl Med Mol Imaging	34	–	No mention	T1; DWI-b1000; ADC; SWI; MRF; 3D T1-Gd; FLAIR	I–IV
[39]	He	2022	BMC Med Imaging	81	–	2/3 T	T1, T1-Gd, T2, DWI	I–IV
[40]	Huang	2021	J Comput Assist Tomogr	59	–	1/3 T	T1, T1-Gd, T2, FLAIR	II–IV
[41]	Huang	2021	Cancer Sci	53	–	1/3 T	T1, T1-Gd, T2, FLAIR	I–IV
[42]	Jiang	2019	Eur J Radiol	122	35	1/3 T	3D T1-Gd, T2	II–III
[43]	Kihira	2021	Neurooncol Adv	111	20	7/1.5 T and 3 T	3D T1-Gd, 3D FLAIR, DWI	II–IV
[44]	Kihira	2022	Cancers (Basel)	208	31	No mention	FLAIR	II–IV
[11]	Korfiatis	2016	Med Phys	155	–	4/1.5 T and 3 T	T1, T1-Gd, T2	IV
[14]	Le	2020	J Pers Med	53	–	TCIA	T1, T1-Gd, T2, FLAIR	IV
[12]	Li	2018	Eur Radiol	193	60	1/3 T	T1, T1-Gd, T2, FLAIR	IV
[45]	Lu	2020	Magn Reson Imaging	181	54	3/1.5 T and 3 T	T1-Gd	IV
[46]	Pasquini	2021	Front Oncol	156	–	2/1.5 T and 3 T	3D T1-Gd, T2, FLAIR, DWI, DSC	IV
[53]	Pease	2022	J Neurooncol	114	28	TCIA	T1-Gd, FLAIR	IV
[47]	Sasaki	2019	Sci Rep	162	–	10/1.5 T and 3 T	T1, T2, T1-Gd	IV
[48]	Shboul	2020	Sci Rep	108	27	TCIA	T1, T1-Gd, T2, FLAIR	II–III
[15]	Sohn	2021	J Neurooncol	418	126	1/3 T	3D T2, 3D FLAIR, 3D T1-Gd	IV
[49]	Verduin	2021	Cancers (Basel)	147	43	Several/1 T–3 T	T1-Gd, T2	IV
[50]	Vils	2021	Front Oncol	118	49	17/1 T–3 T	T1-Gd	IV
[51]	Wei	2019	Eur Radiol	105	31	1/3 T	T1-Gd, FLAIR, ADC	II–IV
[52]	Xi	2018	J Magn Reson Imaging	118	20	2/3 T	T1, T1-Gd, T2	IV

*Abbreviations*: *ADC* apparent diffusion coefficient, *ASL* arterial spin labeling, *DWI* diffusion-weighted imaging, *DSC* dynamic susceptibility contrast, *MRF* magnetic resonance fingerprinting, *T1-Gd* T1-weighted images after gadolinium injection, *TCIA* The Cancer Imaging Archive, *SWI* susceptibility-weighted imaging

### RQS assessment

**Fig. 2:**
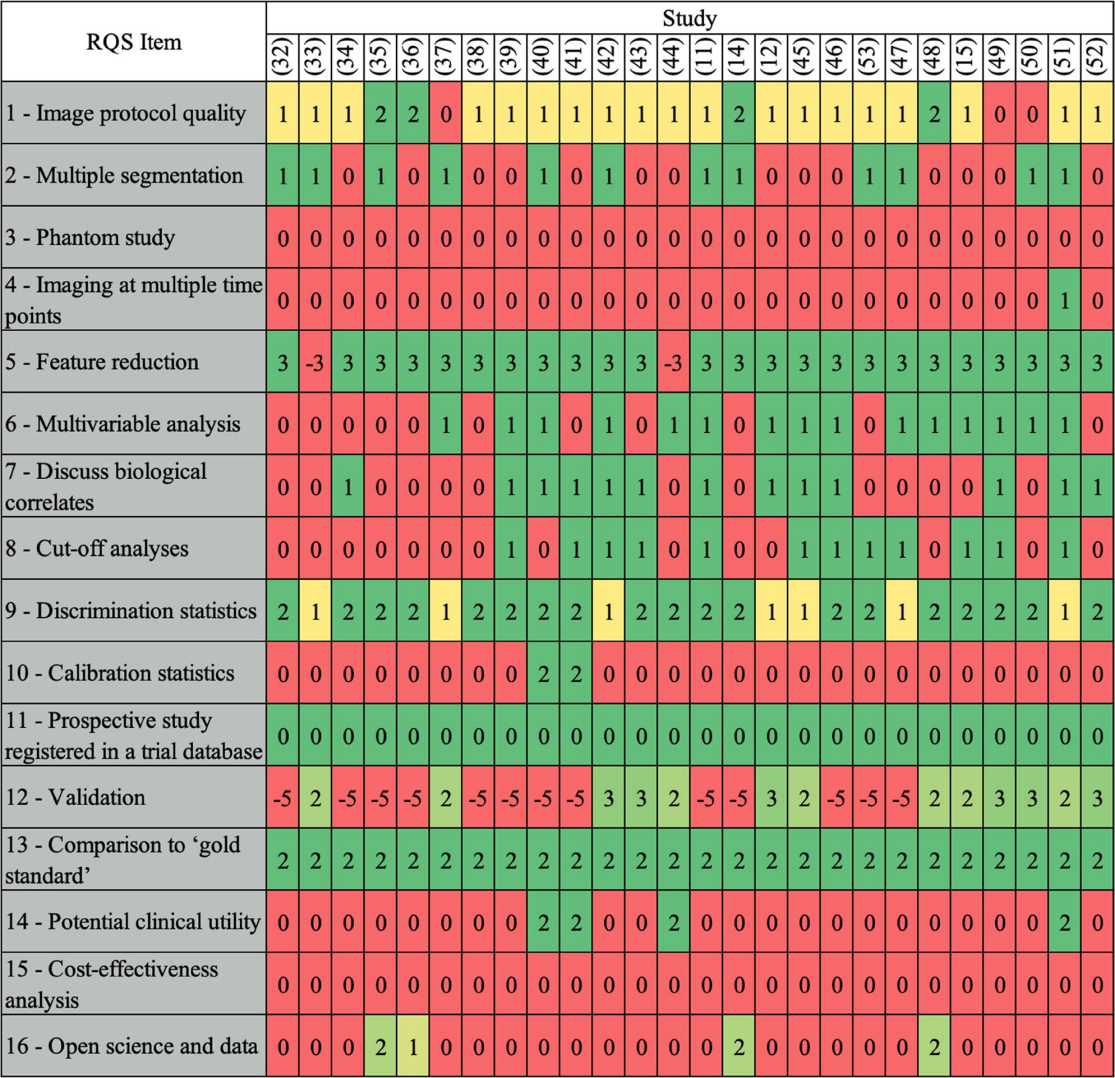
Summary of radiomics quality scores (RQS) assessment results of the 26 included studies per domain. Each row of the plot shows the distribution of the scores achieved by the studies for a domain. Colors from red to green denote progressive increase from minimum to maximum score obtainable for each item. Abbreviations: RQS, Radiomics Quality Score

Results of the RQS assessment for the 26 included studies are summarized in Table 3 and shown in Fig. 2. The median total score was 8 (22%), ranging between 3 (8%) and 16 (44%), from the maximum RQS score of 36 (100%).

**Table 3:** RQS results according to the six domains and overall, along with the classification accuracy achieved for the MGMT methylation status prediction with models based only on radiomic features

Ref	First author	RQS							AUC
		Domain						Total score (%)	
		1	2	3	4	5	6		
[32]	Calabrese E	2	−2	2	2	0	0	4 (11)	0.70
[33]	Chen S	2	−1	2	1	0	0	4 (11)	0.90
[34]	Crisi G	1	−2	3	2	0	0	4 (11)	0.84
[35]	Do DT	3	−2	2	2	0	2	7 (19)	0.87
[36]	Hajianfar G	2	−2	2	2	0	1	5 (14)	0.78
[37]	Haubold J	1	5	3	1	0	0	10 (28)	0.74
[38]	Haubold J	1	−2	2	2	0	0	3 (8)	0.76
[39]	He J	1	−2	4	3	0	0	6 (17)	0.83
[40]	Huang WY	2	−2	6	4	0	0	10 (28)	0.86
[41]	Huang WY	1	−2	5	5	0	0	9 (25)	0.83
[42]	Jiang C	2	6	4	2	0	0	14 (39)	0.90
[43]	Kihira S	1	6	3	3	0	0	13 (36)	0.70^a^
[44]	Kihira S	1	−1	5	2	0	0	7 (19)	0.62^a^
[11]	Korfiatis P	2	−2	4	3	0	0	7 (19)	0.85
[14]	Le NQK	3	−2	2	2	0	2	7 (19)	0.90
[12]	Li ZC	1	6	4	1	0	0	12 (33)	0.88
[45]	Lu Y	1	5	4	2	0	0	12 (33)	0.62^a^
[46]	Pasquini L	1	−2	4	3	0	0	6 (17)	0.69
[53]	Pease M	2	−2	2	3	0	0	5 (14)	0.92
[47]	Sasaki T	2	−2	3	2	0	0	5 (14)	0.71
[48]	Shboul ZA	2	5	3	2	0	2	14 (39)	0.70
[15]	Sohn B	1	5	3	3	0	0	12 (33)	0.65
[49]	Verduin M	0	6	4	3	0	0	13 (36)	0.63
[50]	Vils A	1	6	3	2	0	0	12 (33)	0.67
[51]	Wei J	3	5	6	2	0	0	16 (44)	0.90
[52]	Xi YB	1	6	3	2	0	0	12 (33)	0.80^a^

Domain 1: protocol quality and stability in image and segmentation (0–5 points); domain 2: feature selection and validation (−8 to 8 points); domain 3: biologic/clinical validation and utility (0–6 points); domain 4: model performance index (0–5 points); domain 5: High level of evidence (0–8 points); domain 6: open science and data (0–4 points)

*cv* cross-validation, *AUC* area under the curve, *RQS* Radiomics Quality Score, *MGMT* O6-methylguanine-DNA methyltransferase

^a^AUC was not available for this study and accuracy is reported instead

In domain 1, most studies (23/26, 88%) provided a sufficiently detailed description of the image protocol, and only four (15%) used a public protocol. Multiple segmentations by different physicians were done in twelve studies (46%). Phantom studies were never conducted.

In domain 2, all studies but two (92%) performed feature selection. Thirteen out of 26 studies (50%) performed validation without retraining the proposed model (seven based on a dataset of the same institute, and six using an external dataset), and the remaining 13 (50%) did not validate the trained model on a separate dataset.

In domain 3, the majority of studies (15/26, 58%) performed multivariable analysis with non-radiomic features. Biological correlates were discussed in 13 out of 26 (50%) studies. Comparison to gold standard was done in all included studies. Of note, we considered “gold standard” several types of MGMT methylation status analysis, as there is no consensus on the best assay to use [54] and high variability between threshold of MGMT methylation values [55]. Only four studies (15%) performed quantitative analysis (e.g., decision curve analysis) to report on the potential clinical utility.

In domain 4, all studies reported discrimination statistics (AUC or accuracy) along with their statistical significance (*p* values or confidence intervals) and most of them (19/26, 73%) computed those statistics using a resampling method (e.g., bootstrapping, cross-validation); some performed also cut-off analysis (12/26, 46%). In contrast, only two studies (8%) reported calibration statistics.

In domain 5, all studies had a retrospective design; no study performed a prospective validation of the radiomics signature in an appropriate trial, nor a cost-effectiveness analysis.

In domain 6, only four studies had open-source scans (15%) and three of them used also open-source segmentations of regions of interest [56].

We did not observe a remarkable difference in RQS scores when comparing more recent studies to past works (Supplementary Figure 1).

### TRIPOD assessment

The adherence to all TRIPOD items is reported in Supplementary Table 2 for each study. The median total score was 15.5 (range 10–23). The 26 studies met between 31% and 68% of the TRIPOD items, considering only the applicable items. TRIPOD items from the background, study design, eligibility criteria, outcome assessment blinding, definition and handling of the predictors, model development, limitations, and interpretation were reported in more than 80% of the studies (Table 4). Lower percentages (between 60% and 80%) of adherence to items related to key study dates and setting, times and methods of outcome definition, blinding of the predictors’ assessment, and implications were obtained. Instead, other TRIPOD items from title and abstract, sample size, statistical analysis methods, participants, model specification and performance, and supplementary information were reported in less than 30% of the studies. No study performed model updating.

**Table 4:** Summary of TRIPOD adherence of the 26 included studies

TRIPOD section	TRIPOD item	Reported		Not reported		Not applicable	
		*n*	%^a^	*n*	%^a^	*n*	%
Title and abstract	1	0	0%	26	100%	0	0
	2	0	0%	26	100%	0	0
Background and objectives	3a	24	92%	2	8%	0	0
	3b	13	50%	13	50%	0	0
Source of data	4a	25	96%	1	4%	0	0
	4b	19	73%	7	27%	0	0
Participants	5a	18	69%	8	31%	0	0
	5b	24	92%	2	8%	0	0
	5c	0	-	0	-	26	100%
Outcome	6a	20	77%	6	23%	0	0
	6b	25	96%	1	4%	0	0
Predictors	7a	22	85%	4	15%	0	0
	7b	17	65%	9	35%	0	0
Sample size	8	7	27%	19	73%	0	0
Missing data	9	14	54%	12	46%	0	0
Statistical analysis methods	10a	26	100%	0	0%	0	0
	10b	2	8%	24	92%	0	0
	10c	1	8%	12	92%	13	50%
	10d	1	4%	25	96%	0	0
	10e	0	-	0	-	26	100%
Risk groups	11	3	60%	2	40%	21	81%
Development vs validation	12	7	54%	6	46%	13	50%
Participants	13a	7	27%	19	73%	0	0
	13b	6	23%	20	77%	0	0
	13c	2	15%	11	85%	13	50%
Model development	14a	22	85%	4	15%	0	0
	14b	0	0%	1	100%	25	96%
Model specification	15a	2	8%	24	92%	0	0
	15b	1	4%	25	96%	0	0
Model performance	16	2	8%	24	92%	0	0
Model updating	17	0	-	0	-	26	100%
Limitations	18	23	88%	3	12%	0	0
Interpretation	19a	12	92%	1	8%	13	50%
	19b	26	100%	0	0%	0	0
Implications	20	19	73%	7	27%	0	0
Supplementary information	21	3	12%	23	88%	0	0
Funding	22	10	38%	16	62%	0	0

*TRIPOD* Transparent Reporting of a multivariable prediction model for Individual Prognosis or Diagnosis

^a^Calculated over the applicable items only

We did not observe a remarkable difference in TRIPOD scores when comparing more recent studies to past works (Supplementary Figure 1).

### QUADAS-2 assessment

Results of the QUADAS-2 assessment are illustrated in Supplementary Figure 2 and reported in detail in the Supplementary Results. Briefly, overall risk of bias was low in 10 studies, unclear in one, and high in 15; as for the applicability concern related to the present review question, it was low in all but one of the studies.

### Classification accuracy and methodology quality

**Fig. 3:**
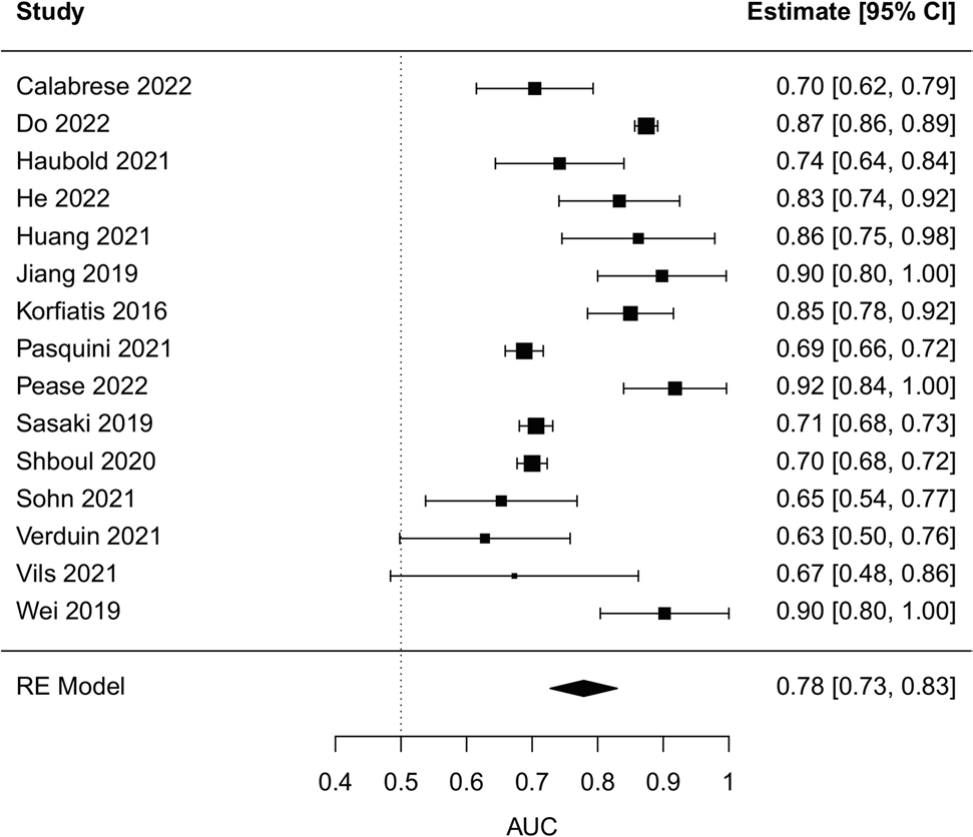
Forest plot of radiomic studies with available data on the AUC and its uncertainty. The estimate of the pooled AUC based on the random effect model is reported on the last line of the plot. Abbreviations: RE, Random Effect; AUC, area under the curve

**Fig. 4:**
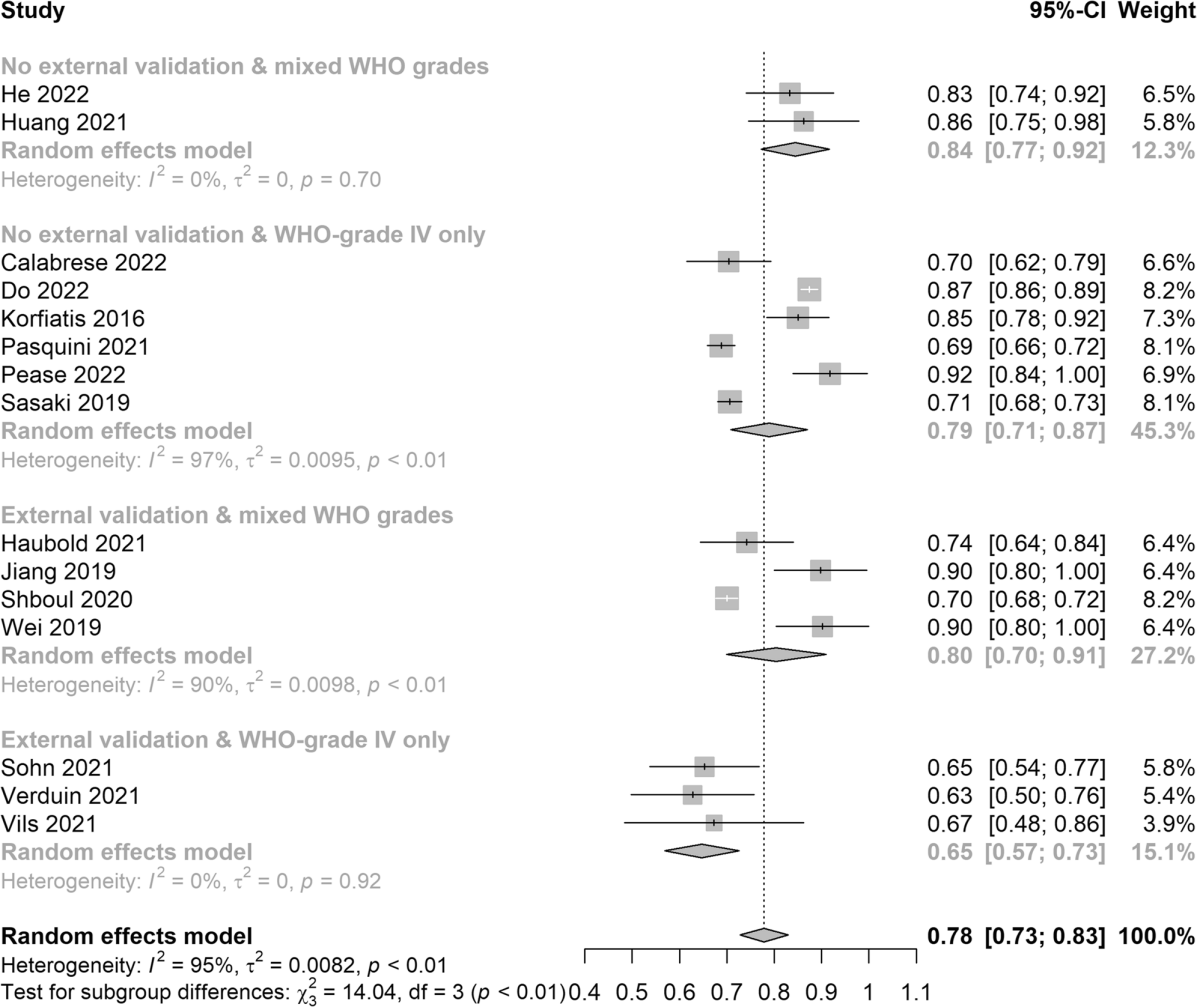
Forest plots with the results of the subgroup analysis. Studies were grouped based on the combination of two factors: (1) whether an external validation was performed; (2) whether WHO-grade IV glioma were only included in their analysis. Abbreviations: AUC, area under the curve; SE, standard error; CI, confidence interval

In total, 13 studies provided sufficient information in the text to compute the pooled AUC. As for the other 13, only three provided such missing data following our email request [32, 35, 42]. Therefore, 16 studies were considered for meta-analysis. However, we found that two studies [40, 41] included the same cohort of patients recruited in the same center during the same period, and therefore only one of these two studies (the one with the highest number of patients) was finally incorporated in the meta-analysis [40] . The pooled AUC of the 15 studies was estimated to be equal to 0.778 (95% CI 0.728–0.830, *I*^2^ = 94.08%) (Fig. 3). Subgroup analysis indicated that studies with external validation and including only patients with WHO-grade IV tumors had AUC values significantly lower (0.647, 95% CI 0.569–0.726, *I*^2^ = 0%) than others (test for subgroup differences: *χ*^2^ = 14.04, df = 3, *p* = 0.0029) (Fig. 4). A meta-regression model of the AUCs based on predictors such as RQS and TRIPOD total scores was not globally significant (*χ*^2^ = 0.8506, df = 2, *p* = 0.6536). After excluding each study one at the time and recomputing the pooled AUC, there was no study that contributed significantly to the heterogeneity observed in this sample (Supplementary Figure 3). Publication bias was absent according to visual inspection of the funnel plot which did not suggest substantial asymmetry (Supplementary Figure 4), as confirmed also by Egger’s test (*t* = −0.15, df = 13, *p* = 0.8823).

Of note, the above results were obtained with models based on radiomic features only. There were 13 out of 26 studies investigating multivariable models integrating radiomic with non-radiomic features and comparing AUCs with models based only on radiomics: in seven of them [11, 12, 37, 40, 46, 47, 50], non-radiomic features were not selected by the algorithm and therefore they did not improve model accuracy; in other four studies, AUC values increased when the model included non-radiomic features, such as other radiological features of the images [39], Visually AcceSAble Rembrandt Images (VASARI) features [45, 49], fractal features [48], or prediction results obtained by a multi-label classification model [15]; in the remaining two studies, AUC values were lower when age [42] or apparent diffusion coefficient values of tumor/edema areas [51] were included in a multivariate model.

## Discussion

We reviewed 26 studies aiming to predict MGMT promoter methylation in patients with glioma based on MRI-radiomic features. We analyzed the adherence of these studies to the RQS and TRIPOD guidelines, finding generally unsatisfactory results for the two scales and heterogeneous classification performances. We performed a meta-analysis on the classification performances obtaining a pooled AUC of 0.78 and finding significantly lower AUC value (0.65) for studies performing external validation only on grade IV gliomas.

### RQS assessment

In our review, the median total RQS score of the analyzed studies was globally low (8/36 points, 22%), in agreement with other methodological reviews of radiomic studies [18–20, 26, 27, 57].

Regarding the specific RQS domains, most studies complied with domain 3 (biologic/clinical validation and utility), where 19 out of 26 studies obtained at least a score of 3 out of 6. However, a perfect score in this domain was rarely achieved due to the lack of a decision curve analysis. Such analysis enables estimation of the clinical net benefit achievable by the prediction/diagnostic model, although it is rarely performed in medical literature [58]. It should also be noted that this tool is often misused in literature, even though it can still provide useful insights [59].

Referring to domain 1 (protocol quality and stability in image and segmentation), less than half of the analyzed studies (12/26) did multiple segmentations: this item is very time-consuming, but it increases the reproducibility of the results [17, 60].

In domain 4 (model performance index), all studies performed discrimination analysis reporting an appropriate accuracy metric and most of them also used resampling techniques (bootstrap or cross-validation) to reduce the overfitting issue. However, calibration (which measures the agreement between the probability of being classified as positive and the true underlying risk of being positive) was rarely conducted, probably because this analysis is not commonly performed outside the machine learning community. Future radiomic studies should also consider this analysis when individual predictions are made and used to support clinical decision-making.

As reported by previous works [18, 26], the RQS is inherently inferior in retrospective and radio-genomic correlation studies, such as those included in this review. They inevitably performed poorly in domain 5 (high level of evidence), in which 7 out of 36 total points are attributed if the study is prospective. Moreover, apart from studies that used large public databases [12, 14, 48], no work shared their images, obtaining low scores in domain 6 (open science and data). Therefore, 11 RQS points out of 36 coming from domains 5 and 6 were difficult to attribute, suggesting that some RQS items may be too strict for most studies as recently highlighted [61].

In domain 2 (feature selection and validation), almost all studies performed a feature reduction step. However, half of them did not perform the validation step (−5 points), because they did not test the final model on a separate dataset. This has serious implications on the classification performance of the models.

### Classification performance

Unsurprisingly, studies without external validation performed better than those with a separate test set, according to our meta-analysis. Indeed, machine learning methods are prone to overfitting to the trained dataset, especially when a feature selection step is performed before training the model [62]. This is the reason why most recommended guidelines such as TRIPOD [22] and RQS require a step of external validation after developing the prediction model [60]. Without this step, it is impossible to reach generalizable results. Therefore, results of studies suffering from this issue should be interpreted carefully.

Our meta-analysis proved that MGMT promoter methylation prediction was less accurate when considering a homogeneous cohort of patients with grade IV gliomas only, whose radiological characteristics are similar. In other words, radiomic models considering heterogeneous glioma grades perform better because they may be influenced by the different levels of MGMT promoter methylation between lower- and higher-grade gliomas [63].

Other sources of variability may be found in the different choices of tumor segmentation. Although specific tumor compartments were identified in 19 studies (as shown in Supplementary Table 1), they were not standardized across studies and therefore subgroup analysis was not possible. Moreover, the indication of contouring the “whole tumor” made by some studies was not always sufficiently specific to understand the precise extension of the tumor considered (i.e., if edema or necrosis was included). Future studies should provide more details of the tumor compartments considered (possibly illustrating representative examples) and are encouraged to develop separate radiomic models on each compartment as well as on their union to increase comparability and reproducibility across studies.

Further sources of variability were identified in the sequences used to extract radiomic features. Only a small number (5 out of 26) of studies used public databases, while the majority relied on retrospective data collected in single centers, which demonstrated a substantial heterogeneity. For instance, only 6 studies employed 3D-T1-weighted imaging sequences post-contrast, while 13 and 18 studies made use of 2D-T1-weighted imaging sequences without and with contrast, respectively; there were 14 studies considering 2D-T2-weighted imaging sequences and only 2 employing 3D-T2-weighted imaging sequences. This substantial heterogeneity of the conventional sequences markedly diminishes the generalizability of the findings. Standardizing the sequences across various centers (including prospective studies) and enlarging the number of cases in public databases can alleviate this variability.

Our review relied on conventional radiomic studies involving hand-crafted features, which offer a potentially high level of interpretability and may be appropriate for relatively small datasets, such as those commonly gathered in neuro-oncological research. On the other hand, deep-learning techniques are increasingly being employed to automatically extract radiomic features that have the potential to capture complex, high-dimensional patterns within the data. However, more recent studies using deep-learning radiomics to predict MGMT promoter methylation status reported heterogeneous results, obtaining high [33, 64] and low [65] classification performances. Thus, it appears that deep-learning studies may also be affected by comparable concerns as those identified in this review. However, further evidence is required to conduct a more thorough investigation of this matter.

### Risk of bias

Most of the included studies had high or unclear risk of bias (16/26), as estimated through QUADAS-2 tool: this was partially in agreement with the results of the RQS and TRIPOD, which indicated even lower methodological quality overall. This is because RQS and TRIPOD examine the issues related to several methodological choices in greater depth and specificity than QUADAS-2 for studies that develop radiomic and prediction models.

### Limitations

One limitation of this study was that only papers written in English were included. Moreover, gray literature was not incorporated; nevertheless, we believe the included studies provided a comprehensive representation of the literature, as we found no evidence of publication bias (Supplementary Figure 4). Another limitation was that certain studies included in the systematic review did not report AUC values with uncertainty measures, and consequently could not be included in the meta-analysis.

## Conclusions

Adherence of the published articles to RQS items or the indications of TRIPOD was generally low. Radiomic models do not provide accurate predictions of the MGMT promoter methylation status in grade IV gliomas. Therefore, to date, they are not ready to be integrated into clinical practice. Future studies aiming to predict MGMT promoter methylation status with radiomics should include homogeneous cohorts of glioblastoma patients and have a sufficiently large number of cases to permit a proper external validation; adherence to current reporting guidelines and radiomic pipelines (such as RQS, TRIPOD, and CLEAR [17, 22, 61]) should also be increased to improve quality, reliability, and, therefore, inter-study comparability.

### Supplementary Information

Below is the link to the electronic supplementary material.

Supplementary file1 (PDF 1642 KB)

## Abbreviations

AUC: Area Under the Curve
IBSI: Image Biomarker Standardization Initiative
MGMT: O6-Methylguanine-DNA methyltransferase
QUADAS-2: Quality Assessment of Diagnostic Accuracy Studies 2
RQS: Radiomics Quality Score
TRIPOD: Transparent Reporting of a Multivariable Prediction Model for Individual Prognosis Or Diagnosis

## Declarations

### Guarantor

The scientific guarantor of this publication is prof. Luca Maria Sconfienza.

### Statistics and biometry

One of the authors has significant statistical expertise.

### Study subjects or cohorts overlap

Since this study is a systematic review and meta-analysis, all data were collected from previously published studies.

### Methodology

• Systematic review and meta-analysis
