# Supplementary material for: Quality assessment of the MRI-radiomics studies for MGMT promoter methylation prediction in glioma: a systematic review and meta-analysis
Source: Eur Radiol. 2024 Feb 3;34(9):5802–15. doi: 10.1007/s00330-024-10594-x (PMC11364578; doi:10.1007/s00330-024-10594-x)
Supplement: Supplementary file 1 — Supplementary file1 (PDF 1642 KB) [file 330_2024_10594_MOESM1_ESM.pdf]

Supplementary materials

Supplementary Methods

Two reviewers (F.M.D. and R.P.) assessed whether the following four domains of QUADAS-2 could have introduced bias in the included studies: (1) patient selection — description of how patients were recruited (use of consecutive/random sample, avoidance of case-control design, appropriate exclusion criteria); (2) index test — how the index test (i.e., radiomics-based model prediction) was conducted and interpreted, in particular whether an external validation was performed; (3) reference standard — how the reference test (i.e., MGMT methylation status analysis) was conducted and interpreted; and (4) flow and timing — whether all patients had the index or reference test and were included in the final analysis. The first three domains were also assessed for possible concerns related to their applicability to the review question. The domains related to the risk of bias and to the applicability concerns were examined for each study and categorized as either low risk, high risk, or unclear. An overall assessment of the risk of bias and, separately, of the applicability concerns was also conducted based on the results of the corresponding domains in each study. If at least one domain had "high risk of bias", then overall risk was categorized as "high". If at least two domains had "unclear risk of bias", then overall risk was categorized as "unclear". The same criteria were used for overall applicability concerns. The same reviewers independently reviewed each study and any disagreement was resolved by consensus.

Supplementary Results

The results of the QUADAS-2 assessment are illustrated in Supplementary Figure 1. Overall risk of bias was low in 10 studies, unclear in one, and high in 15. In particular, one study had high risk of bias in domain 1 (patient selection) because of the enrollment of patients with recurrent glioblastoma only [50]. Other 13 studies had high risk of bias in domain 2 (index test) because they did not perform an external validation [11, 14, 32, 34–36, 38–41, 46, 47, 53]. One work [42] had high risk of bias in domain 4 (flow and timing) because of the use of different methods for MGMT methylation analysis among patients in the training and validation sets and to the random selection of a small validation set of patients from a public dataset. Other studies had unclear risk of bias in domain 2 (index test), because it was not specified whether a pre-determined threshold/cut-off was used for the model predictions to classify positive results; and in domain 4 (flow and timing) an unclear risk of bias was identified because in some studies the reference standard was not available for all patients.

Overall, the majority of studies (25 out of 26) had low applicability concerns related to the present review question. One study [34] had high applicability concerns, because radiomic features were extracted only from perfusion dynamic susceptibility contrast MR images, while such process was done on conventional images (T1, T2, and/or FLAIR) in all the other studies. Regarding domain 3

(reference standard), we acknowledge that no international consensus on MGMT methylation method and threshold is recognized [55]. Nevertheless, we searched which method was utilized to assess MGMT methylation status: 20 studies mentioned a specific method whereas six did not. As for the threshold, only six studies reported a value to discriminate between positive and negative results [34, 41, 42, 47, 51, 52] and, therefore, these were classified as having low applicability concerns for domain 3; all the other 20 studies were classified as having unclear applicability concerns for domain 3.

Supplementary Table 1. Additional information of the studies included in this review.

Study	Glioma grades	Other explored markers	Tumor comparment considered	Tumor compartments whose features were selected	MRI sequences whose features were selected	Total number of features extracted	Methods used for feature selection	Methods used for classification
Calabrese 2022 [32]	IV	IDH, TERT, TP53, PTEN, ATRX, CDKN2A/B, EGFR, aneuploidy of ch7 and ch10	CE, NEC, HYP	no mention	no mention	5300	Three-step process with a 5-fold cross-validation approach:<br>1) Univariate feature selection with mutual information (1024 best correlated features were selected);<br>2) recursive feature elimination with a random forest classifier to rank the best features for each cross-validation fold<br>3) the 32 features with the best average rank across folds were finally selected	Random Forest, Convolutional Neural Network
Chen 2022 [33]	65 GBM and 46 LGG	-	CE+NEC, CE+NEC+HYP	no mention	no mention	688	No feature selection	Convolutional Neural Network (ResNet-18)
Crisi 2020 [34]	IV	-	CE+NEC	no compartments	rCBV (4), rCBF (1)	92	Mann-Whitney test with Bonferroni correction	Naive Bayes, Decision Trees, Multilayer Perceptron
Do 2022 [35]	IV	-	CE, NET, HYP	no mention	all sequences (14)	704	XGBoost and Genetic Algorithm	Random Forest, Extreme Gradient Boosting, Support Vector Machine
Hajianfar 2019 [36]	IV	-	whole tumor, CE, NEC, HYP	whole tumor, edema, necrosis, active (enhanced tumor)	T1Gd, FLAIR	8519	Different feature selection methods and their combinations:<br>(1) select K best;<br>(2) mutual information regression;<br>(3) select from model;<br>(4) select percentile;<br>(5) variance threshold	AdaBoost, Bagging Decision Tree, Naive Bayes, Decision Tree, Gaussian Naive Bayes, K-Nearest Neighbors, Logistic Regression, Multilayer Perceptron, Quadratic Discriminant Analysis, Random Forest, Stochastic Gradient Descent, Support Vector Machine
Haubold 2021 [37]	28 LGG; 187 HGG	grade, ATRX, 1p19q, IDH	not specified	no compartments	not mentioned	1562	Boruta	Extreme Gradient Boosting
Haubold 2020 [38]	1 grade I; 13 grade II; 7 grade III; 9 grade IV	1p19q, ATRX, IDH, grade	whole tumor	no compartments	T1, T1Gd, DWI (b1000)	19284	t-score; f-score (ANOVA); chi-square; LCSI; randomized logistic regression repeated 200 times	Random Forest, Support Vector Machine
He 2022 [39]	2 grade I; 26 grade II; 29 grade III; 24 grade IV	IDH, TERT, 1p/19q	HYP	HYP	T2, T1, DWI (b1000), T1Gd	107	LASSO	Logistic Regression

Huang 2021 [40]	14 LGG; 45 HGG	grade, IDH	whole tumor	no compartments	not mentioned	369	Univariate statistical tests, LASSO	Logistic Regression
Huang 2021 [41]	I-IV	-	whole tumor	no compartments	T1, T1Gd, T2, FLAIR,	396	minimum redundancy maximum relevance; top five features most correlated with MGMT status were selected	Logistic Regression
Jiang 2019 [42]	II-III	-	CE+HYP	no compartments	3D-T1Gd (4); T2 (11)	1702	LASSO	AdaBoost, Random Forest, Support Vector Machine
Kihira 2021 [43]	7 grade II; 12 grade III; 92 grade IV	IDH, EGFR, ATRX, PTEN, TP53	whole tumor	no compartments	3D-T1Gd (1); 3D-FLAIR (1)	368	LASSO	Logistic Regression
Kihira 2022 [44]	124 GBM and other 84 LGG	IDH	whole tumor	FLAIR	whole tumor	95	No selection	Multilayer Perceptron, Random Forest, Extreme Gradient Boosting, Support Vector Machine
Korfiatis 2016 [11]	IV	-	CE, NET	no mention	T2 (7); T1Gd (4)	not reported	Ridge regression	Random Forest, Support Vector Machine
Le 2020 [14]	IV	-	CE, NET, HYP	CE, NET, HYP	T1, T2, FLAIR	704	F-score; RFE	K-Nearest Neighbors, Naïve Bayes, Random Forest, Extreme Gradient Boosting, Support Vector Machine
Li 2018 [12]	IV	-	NEC, edema, NET, CE	core (2), edema (2), necrosi (1), enhanced area (1)	T1 (2), T2 (2), T1Gd (1), FLAIR (1)	1705	Boruta; Mann-Whitney test with Benjamini-Hochberg correction and Spearman correlation coefficient for redundancy evaluation	Random Forest
Lu 2020 [45]	IV	-	CE, NEC, CE+NEC	CE (5), NEC (2), whole tumor (4)	T1Gd	333	Boruta	K-Nearest Neighbors, Decision Tree, Random Forest, Gradient Boosting Tree, Support Vector Machine, Deep Learning algorithm (not otherwise specified)
Pasquini 2021 [46]	IV	Ki-67, IDH, EGFR	CE, NEC, HYP	CE	FLAIR (top 15)	1871	Boruta	AdaBoost, Extreme Gradient Boosting, Gradient Boosting Tree, Decision Tree, Random Forest, Logistic Regression, K-Nearest Neighbors, ensemble stacking, ensemble stacking with AdaBoost
Pease 2022 [53]	IV	EGFR	CE, NEC, HYP, whole tumor	not mentioned	T1Gd and FLAIR (100)	4880	Maximum Relevance Minimum Redundancy	Support Vector Machine
Sasaki 2019 [47]	IV	-	CE+NEC, HYP	CE+NEC (1), HYP (1)	T1Gd (2)	489	LASSO	Logistic Regression
Shboul 2020 [48]	II-III	IDH, 1p/19q, ATRX, TERT	CE, HYP, NET	not specified for the radiomic model	not specified for the radiomics model	680	Recursive feature selection	Extreme Gradient Boosting

Sohn 2021 [15]	IV	IDH, ATRX, EGFR	CE, HYP	CE	T2 (1)	660	LASSO	Support Vector Machine
Verduin 2021 [49]	IV	IDH, EGFR	CE, tumor+edema	no mention	T2 (3)	1197	Spearman correlation coefficient, lower AUC in univariate ROC analysis; top 20 features ranked by importance as estimated by Random Forest repeated 1000 times	Extreme Gradient Boosting, Random Forest, Logistic Regression
Vils 2021 [50]	IV	-	CE+NEC, peritumoral	CE+NEC	T1Gd (2)	180	PCA and univariate logistic regression; backward selection based on AIC	Logistic Regression
Wei 2019 [51]	II-IV	-	tumor, edema	edema (4), tumor (6)	T1Gd (3), FLAIR (7)	3051	ICC, CCC, dynamic range analysis, Mann-Whitney without correction; minimum redundancy maximum relevance score; backward selection with BIC	Logistic Regression
Xi 2018 [52]	IV	-	CE+NEC+NET	only one compartment	T1 (4), T2 (19)	1665	LASSO	Support Vector Machine

Abbreviations: CE = contrast-enhanced tumor; NEC = necrosis; NET = non-enhancing tumor (excluding edema on T2/FLAIR); HYP = non-enhancing tumor (including edema on T2/FLAIR)

Supplementary Table 2. Evaluation of the TRIPOD checklist items in the 26 included studies.

Study	Title and abstract		Background and objectives		Source of data		Participants			Outcome		Predictors		Sample size	Missing data	Statistical analysis methods					Risk groups	Development vs validation
	1	2	3a	3b	4a	4b	5a	5b	5c	6a	6b	7a	7b	8	9	10a	10b	10c	10d	10e	11	12
Calabrese 2022 [32]	0	0	1	1	1	1	1	1	NA	1	1	1	1	0	1	1	0	NA	0	NA	NA	NA
Chen 2022 [33]	0	0	1	0	1	1	1	1	NA	0	1	1	0	0	1	1	0	0	0	NA	NA	1
Crisi 2020 [34]	0	0	1	1	1	1	1	1	NA	1	1	1	1	0	0	1	0	NA	0	NA	NA	NA
Do 2022 [35]	0	0	1	1	1	1	1	1	NA	0	0	0	1	1	1	1	0	NA	0	NA	NA	NA
Hajianfar 2019 [36]	0	0	1	1	1	1	1	1	NA	1	1	1	1	1	1	1	0	NA	0	NA	NA	NA
Haubold 2021 [37]	0	0	1	1	1	0	0	1	NA	0	1	1	1	0	0	1	0	NA	0	NA	NA	NA
Haubold 2020 [38]	0	0	1	0	1	0	1	1	NA	0	1	1	0	0	1	1	0	0	0	NA	NA	1
He 2022 [39]	0	0	1	1	1	1	0	1	NA	1	1	1	0	0	1	1	0	NA	0	NA	NA	NA
Huang 2021 [40]	0	0	1	1	1	1	1	1	NA	1	1	1	1	0	0	1	0	NA	0	NA	NA	NA
Huang 2021 [41]	0	0	1	1	1	1	1	1	NA	1	1	1	0	0	0	1	0	NA	1	NA	NA	NA
Jiang 2019 [42]	0	0	1	0	1	1	1	1	NA	1	1	1	1	0	1	1	0	0	0	NA	NA	0
Kihira 2021 [43]	0	0	1	0	1	1	0	1	NA	1	1	1	1	0	1	1	0	NA	0	NA	NA	NA
Kihira 2022 [44]	0	0	1	1	1	0	0	0	NA	1	1	1	0	0	0	1	0	0	0	NA	NA	0
Korfiatis 2016 [11]	0	0	0	1	1	1	1	1	NA	0	1	1	1	0	1	1	1	NA	0	NA	NA	NA
Le 2020 [14]	0	0	1	1	1	1	0	1	NA	1	1	1	1	1	1	1	0	NA	0	NA	NA	NA
Li 2018 [12]	0	0	1	0	1	1	1	1	NA	1	1	0	0	0	0	1	1	0	0	NA	NA	0
Lu 2020 [45]	0	0	1	0	1	1	1	1	NA	1	1	1	1	0	1	1	0	0	0	NA	1	0
Pasquini 2021 [46]	0	0	1	1	1	1	1	1	NA	1	1	1	1	0	0	1	0	NA	0	NA	NA	NA
Pease 2022 [53]	0	0	1	0	1	1	1	1	NA	1	1	1	1	1	1	1	0	0	0	NA	NA	1
Sasaki 2019 [47]	0	0	0	0	1	0	1	1	NA	1	1	1	0	0	0	1	0	NA	0	NA	0	NA

Shboul 2020 [48]	0	0	1	0	0	0	0	0	NA	1	1	1	1	1	0	1	0	0	0	NA	NA	0
Sohn 2021 [15]	0	0	1	0	1	1	0	1	NA	1	1	1	1	0	1	1	0	0	0	NA	NA	1
Verduin 2021 [49]	0	0	1	1	1	1	1	1	NA	1	1	1	0	0	1	1	0	1	0	NA	1	1
Vils 2021 [50]	0	0	1	0	1	0	1	1	NA	0	1	0	1	1	0	1	0	0	0	NA	0	1
Wei 2019 [51]	0	0	1	0	1	1	1	1	NA	1	1	0	0	1	0	1	0	0	0	NA	1	1
Xi 2018 [52]	0	0	1	0	1	0	0	1	NA	1	1	1	1	0	0	1	0	0	0	NA	NA	0

Supplementary Table 2 (continued).

Study	Participants			Model development		Model specification		Model performance	Model updating	Limitations	Interpretation		Implications	Suppl. info	Funding	TOTAL
	13a	13b	13c	14a	14b	15a	15b	16	17	18	19a	19b	20	21	22	
Calabrese 2022 [32]	0	1	NA	1	NA	0	0	0	NA	1	NA	1	0	1	1	18
Chen 2022 [33]	0	0	0	0	NA	0	0	0	NA	1	1	1	1	0	1	15
Crisi 2020 [34]	1	0	NA	1	NA	0	0	0	NA	1	NA	1	1	0	1	17
Do 2022 [35]	0	0	NA	1	NA	0	0	0	NA	1	NA	1	1	1	0	15
Hajianfar 2019 [36]	0	0	NA	1	NA	0	0	0	NA	1	NA	1	1	0	0	17
Haubold 2021 [37]	0	0	NA	1	NA	0	0	0	NA	1	NA	1	0	0	1	12
Haubold 2020 [38]	0	0	0	1	NA	0	0	0	NA	1	1	1	1	0	1	15
He 2022 [39]	1	1	NA	1	NA	0	0	0	NA	1	NA	1	1	0	0	16
Huang 2021 [40]	0	0	NA	1	NA	1	1	1	NA	1	NA	1	1	0	0	18
Huang 2021 [41]	0	0	NA	1	NA	0	0	1	NA	1	NA	1	0	0	0	15
Jiang 2019 [42]	1	0	0	1	NA	0	0	0	NA	1	1	1	1	0	1	18
Kihira 2021 [43]	1	1	NA	1	NA	0	0	0	NA	1	NA	1	0	0	0	15
Kihira 2022 [44]	0	0	0	0	NA	0	0	0	NA	1	1	1	1	0	1	12

Korfiatis 2016 [11]	1	0	NA	1	NA	0	0	0	NA	1	NA	1	1	0	0	16
Le 2020 [14]	0	0	NA	1	NA	0	0	0	NA	1	NA	1	0	0	1	16
Li 2018 [12]	0	0	0	1	NA	0	0	0	NA	1	1	1	1	0	0	14
Lu 2020 [45]	1	0	1	1	NA	0	0	0	NA	0	0	1	1	0	0	17
Pasquini 2021 [46]	0	0	NA	1	NA	0	0	0	NA	1	NA	1	0	0	1	15
Pease 2022 [53]	0	1	0	1	NA	0	0	0	NA	1	1	1	1	0	0	19
Sasaki 2019 [47]	0	0	NA	0	NA	0	0	0	NA	1	NA	1	1	1	0	11
Shboul 2020 [48]	0	0	0	0	NA	0	0	0	NA	0	1	1	1	0	0	10
Sohn 2021 [15]	0	1	0	1	NA	0	0	0	NA	1	1	1	0	0	0	16
Verduin 2021 [49]	0	1	1	1	0	1	0	0	NA	1	1	1	1	0	1	23
Vils 2021 [50]	0	0	0	1	NA	0	0	0	NA	1	1	1	1	0	0	14
Wei 2019 [51]	1	0	0	1	NA	0	0	0	NA	1	1	1	1	0	0	17
Xi 2018 [52]	0	0	0	1	NA	0	0	0	NA	0	1	1	1	0	0	12

Supplementary Figure 1. Evolution of studies' RQS (top panel) and TRIPOD scores (bottom panel) grouped per year of publication. Median, maximum and minimum scores are represented; no remarkable variations among years could be observed.

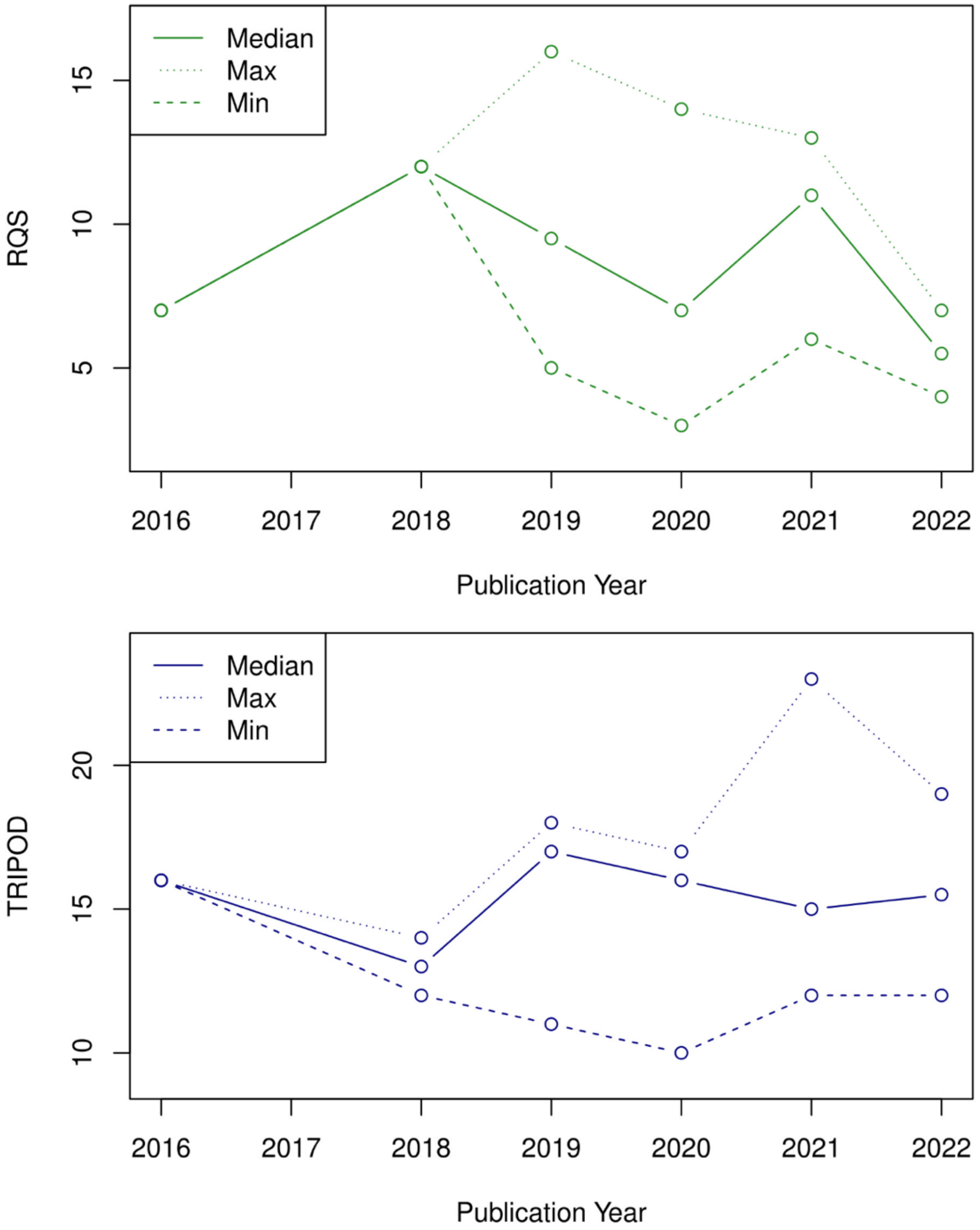

Supplementary Figure 2. Summary of the evaluation of the risk of bias and the applicability concerns in the 26 included studies.

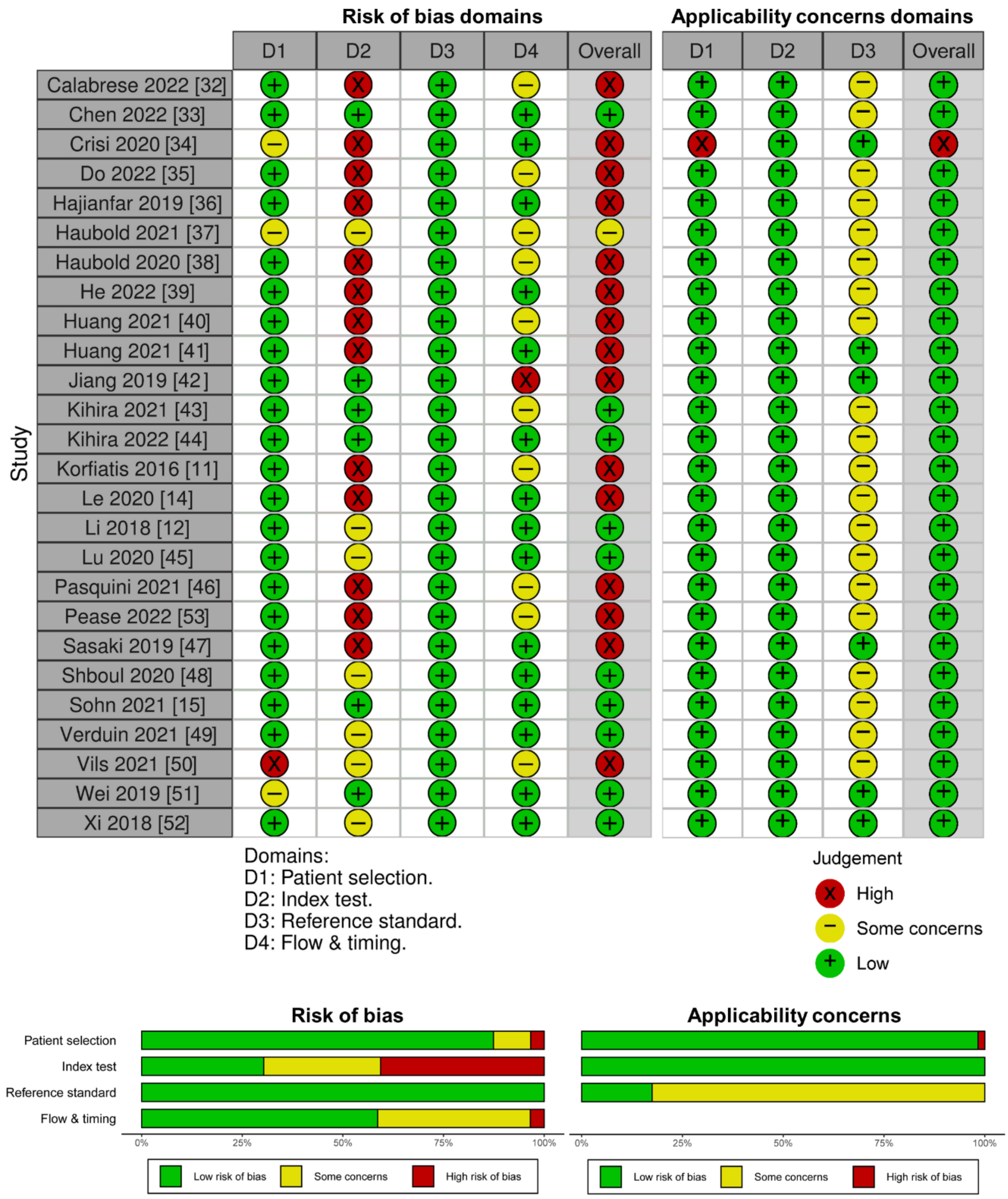

Supplementary Figure 3. Sensitivity analysis of the results of the meta-analysis. Each line illustrates and reports the pooled area under the curve (indicated as Effect Size in the plot, with 95% confidence interval and the heterogeneity statistic I^2) estimated by omitting one specific study at the time from the random-effect model. No study had a significant influence on the model results.

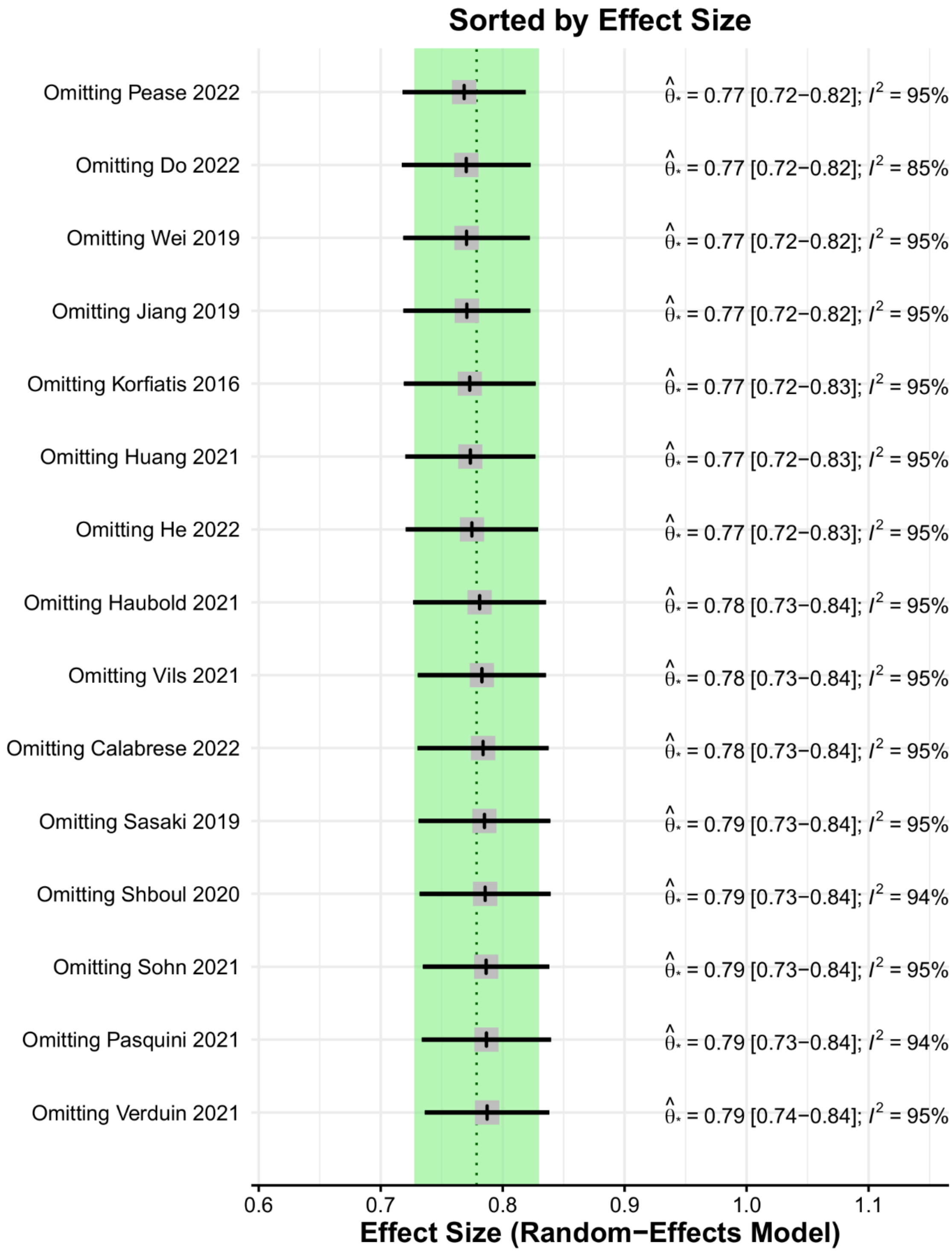

Supplementary Figure 4. Funnel plot to investigate publication bias. No evident asymmetry can be observed, suggesting the absence of publication bias.

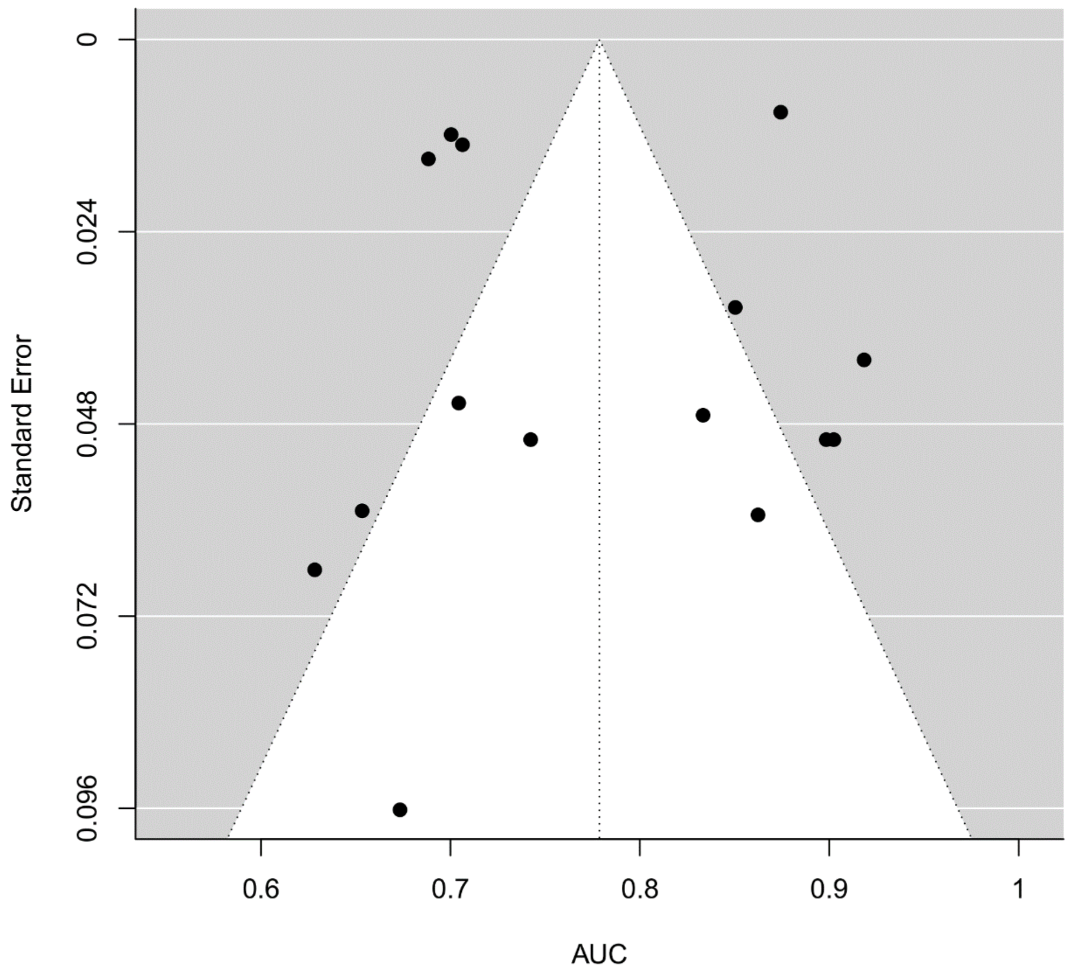

Abbreviation: AUC = area under the curve.
